# A Robust mmWave Radar Framework for Accurate People Counting and Motion Classification

**DOI:** 10.3390/s26041289

**Published:** 2026-02-16

**Authors:** Nuobei Zhang, Haoxuan Li, Adnan Zahid, Yue Tian, Wenda Li

**Affiliations:** 1 School of Engineering and Physical Sciences, Heriot Watt University, Edinburgh EH14 4AS, UK; nz2007@hw.ac.uk (N.Z.); a.zahid@hw.ac.uk (A.Z.); wenda.li@hw.ac.uk (W.L.); 2 School of Opto-Electronic and Communication Engineering, Xiamen University of Technology, Xiamen 361024, China; yue.tian@xmut.edu.cn

**Keywords:** mmWave radar, indoor localization, people counting, machine learning

## Abstract

People counting and occupancy monitoring play a vital role in applications such as intelligent building management, safety control, and resource optimization in future smart cities. Conventional camera and infrared-based methods often suffer from privacy risks, lighting dependency, and limited robustness in complex indoor environments. In this paper, we present a 60 GHz millimeter-wave (mmWave) radar-based occupancy monitoring system that enables accurate and privacy-preserving people counting. The proposed system leverages echo signals processed through Doppler and range spectrogram and analyzed by an enhanced ResNet-50 deep learning model to classify motion states and count individuals. Experimental results collected in a typical indoor environment demonstrate that the system achieves 95.45% accuracy across 6 classes of movements and 98.86% accuracy for people counting (0–3 persons). The method also shows strong adaptability under limited data and robustness to Gaussian blur interference, providing an efficient and reliable solution for intelligent indoor occupancy monitoring.

## 1. Introduction

People counting technology enables the automatic detection of individuals within a defined area, providing critical data for optimizing space utilization, enhancing safety, and improving operational efficiency. It has evolved from simple manual methods to advanced automated systems that leverage sensors, computer vision, and artificial intelligence for real-time, high-accuracy measurements [1].

This capability is increasingly applied across diverse domains, including smart buildings, transportation hubs, retail analytics, and public safety management. Traditional people counting solutions, like visual cameras and infrared sensors, suffer from privacy leakage risk, limited coverage area and high maintenance cost. Furthermore, their performance is susceptible to environmental factors like lighting variations, occlusions, and temperature fluctuations, often leading to reduced accuracy and robustness in real-world operational environments [2].

In comparison, mmWave radar offers a contactless, non-intrusive sensing approach that effectively overcomes many of the limitations of traditional people-counting technologies [3]. By emitting high-frequency electromagnetic waves and analyzing the returned signals, it can accurately capture range, velocity, and subtle motion information of human targets without relying on visual imagery [4]. Its operation is largely unaffected by lighting conditions, temperature variations, or partial occlusions. Consequently, mmWave radar can deliver consistent performance in diverse and dynamic environments [5]. Furthermore, the absence of identifiable visual data promotes privacy protection, making mmWave radar an ideal choice for sensitive enviornment.

Despite the potential of mmWave radar for indoor people counting, several practical limitations remain. First, much of the available hardware was originally designed for automotive or industrial sensing rather than human tracking, which restricts its suitability for this application. For example, paper [6] employed a single TI mmWave radar module for indoor human detection, achieving over 90% sensitivity but suffering from high false alarm rates; only by deploying a second radar did system precision improve from 47% to 98.6%, highlighting the need for complex multi-sensor configurations when using non-specialized equipment [7]. Second, multipath reflections from walls, furniture, and clutter can severely degrade system performance in complex indoor environments. Paper [8] addressed this challenge using an extended-target tracking method with an alpha-extended Kalman filter and density-based clustering to mitigate false associations caused by multipath and target merging, yet the approach still required significant computational resources and careful tuning for specific scenes. Third, there remains a lack of practical, ready-to-deploy solutions that can be easily scaled to real-world settings. Paper [9] proposed an IoT framework integrating multiple mmWave FMCW radars for occupancy monitoring, but coverage limitations due to the range of individual radars and the complexity of network integration restricted its applicability. Collectively, these issues underscore the gap between current research prototypes and scalable mmWave radar systems for indoor people counting.

This paper proposes a novel mmWave radar solution for complex indoor people counting. It operates at 60 GHz with a hybrid online signal processing and offline machine learning framework. The system firstly performs classical radar signal processing to generate range-time and Doppler-time spectrograms. After that, these two spectrograms are fed into an end-to-end neural network for occupancy classification (unmanned/single-person/multi-person with directional motion) with data collected from real-world experiments. The fusion of Doppler and range spectrograms establishes a robust approach to maximizing the information utility of mmWave radar.

The remainder of this paper is structured as follows: Section 2 reviews previous works on people counting; Section 3 presents proposed concepts implemented in our mmWave radar system; Section 4 demonstrates experiment layout and dataset; Section 5 quantifies the experimental results; conclusions are in Section 6.

## 2. Literature Review

Recent advances in mmWave radar technology have enabled its widespread use in human sensing applications [4,10]. Many researchers have focused on leveraging mmWave radar’s high sensitivity to capture range and micro-Doppler features with various platforms, hardware setups and signal processing [11,12].

The work [13] leverages the Novelda X4M03 IR-UWB radar and achieves an accuracy of 92.83% for wide-area counting of 0–10 individuals. It combines both IR imaging and radar returns, with a focus on range domain and energy-based features in the frequency domain. The work relies on the improved CLEAN algorithm and multi-threshold (MT) to distinguish the human number based on power spectral density for both range and frequency. However, such work does not rely on range or Doppler spectrogram; therefore, it cannot provide a trajectory for each individual target. Furthermore, the distribution of energy feature is not a reliable source for detection, as it depends on the size, distance and wearing of the target.

Another work [14] utilizes the TI IWR6843AOP MIMO radar for radar imaging and contains three transmitters and four receivers (a total of 12 vital antennas). It achieves a high resolution of 7.3 cm via spatial boundary sensing technology, with a human counting accuracy of 85.63%. Yet, this work requires significant computational power, as it computes the point cloud, which makes it unsuitable for real-time processing. Additionally, its performance is confined to scenarios involving 1–4 people moving in the same direction, as it depends on static boundary estimation (requiring pre-screening of 0/1 person data). The boundary learning process directly discards multi-person interaction data, resulting in performance downgrade in handling reverse/random movements, which potentially interrupts the detection when individuals were blocked by others.

Low-cost mmWave radar devices can provide cost-effective solutions for real-world application, which is another area worth further exploration. Taking the Infineon BGT60TR13C FMCW radar as an example, ref. [15] achieves an accuracy of 89% in intra-sensor tests through knowledge distillation with a camera sensor. However, it requires cross-modal information from a camera sensor to achieve sufficient accuracy. Such work could perform poorly if the signature in the Doppler and range spectrogram differed due to room layout and change in target shape [16]. In terms of generalization, during cross-sensor tests (same-model radars at different positions and viewing angles), accuracy drops sharply from 89% to 71%, indicating poor adaptability to hardware installation variations and environmental changes. Moreover, this study does not explore opportunities to enhance resolution (theoretical resolution is approximately 15 cm based on parameters of the 60.5–61.5 GHz frequency band and 128 samples/chirp) nor quantify performance in noisy environments, restricting its deployability in actual complex real-world environments.

Meanwhile, some studies report high accuracy but often rely on scenario simplification or dedicated algorithm designs. For instance, work [17], based on the NVA-R661 IR-UWB radar, can achieve 99.9% counting accuracy in queuing scenarios, but its core limitation is that it only applies to simple linear one-way queues (up to three people). Due to the single movement pattern and relatively simple queuing scenarios, this work is not designed to handle signal aliasing from overlapping positions and interleaved trajectories when there are multi-person multi-directional movements (e.g., interactions, reverse, or random directions) [18]. Altogether, it limits its extension to more complex indoor environments.

The system proposed in [19], using a Novelda X4M03 UWB radar, can achieve 95.5% accuracy in moving target counting. It categorizes movement states roughly into static and dynamic by using the range spectrogram only. However, it does not distinguish directions, consequently failing to capture feature differences arising from direction changes in multi-person interactions. This leads to notable limitations in scenarios when precise direction recognition is needed. Work [19] oversimplifies human movement into only two states. It may work less effectively when the monitoring scenario becomes complex and uncontrolled.

Table 1 comprehensively summarizes hardware, signal processing and performance from above works.

Comparing to previous works, the following contributions are made by this paper:

- Different from previous work, our proposed system simultaneously utilizes both Doppler and range spectrograms. Together, these two spectrograms provide better spatial and motion dynamics for more accurate people counting and motion classification.
- A compact 60 GHz FMCW mmWave radar system was developed for real-time indoor occupancy monitoring. A robust signal processing pipeline was implemented to generate spectrograms for machine learning. The system achieves reliable detection within an effective range of up to 8 m under typical indoor environments.
- By leveraging the adaptive learning capabilities of ResNet-50 network model, the framework dynamically adapts to environmental noise and motion state variations. Data augmentation had been deployed to deal with the small size of dataset.
- Multiple experiments were conducted to verify proposed concepts. Experimental results indicate 95.45% accuracy for joint motion-state recognition and 98.86% accuracy for people counting.

## 3. Methodology Radar Signal Processing

**Figure 1:**
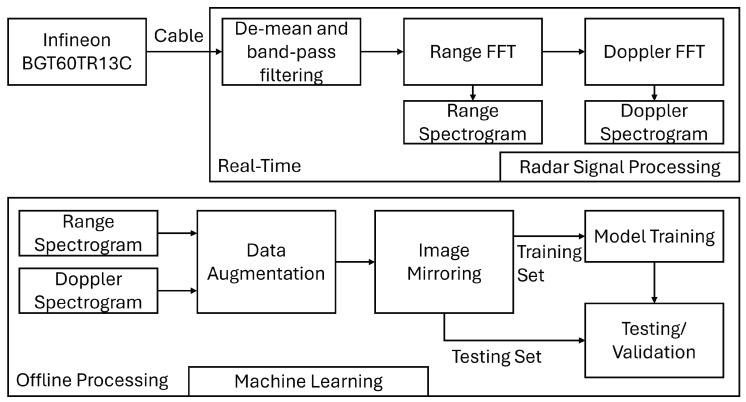
Block diagram of proposed system.

The block diagram of the overall signal processing algorithm is shown in the following Figure 1. As can be seen, there are two main parts in this work, radar signal processing and machine learning. Data from the mmWave radar sensor (Infineon BGT60TR13C) was passed to a laptop through a data link connection. The radar signal processing is implemented in MATLAB R2023b and run in real time on the laptop. This low-complexity signal processing method design also helps the system maintain excellent real-time performance. The testing and training for machine learning models are performed offline.

### 3.1. Radar Signal Processing

The mmWave radar chip Infineon BGT60TR13C [21] transmits a FMCW wave centered at 60 GHz. An ideal FMCW chirp can be characterized by its start frequency $f_{c}$, bandwidth *B*, and chirp duration $T_{s}$ [22]. The complex baseband representation of the transmitted signal $x_{Tx}\left(t\right)$ is given by

(1) $$x_{Tx}\left(t\right)=A_{T}\;\cdot \;\exp\left(j2\pi \left(f_{c}t+\frac{\alpha }{2}t^{2}\right)\right),\;0\leq t\leq T_{s}$$

where $A_{T}$ is the amplitude, $\alpha =B/T_{s}$ is the chirp rate, and *t* is the fast time within a chirp. This model captures the linear frequency sweep of the FMCW signal.

After the Analog-to-Digital (ADC) procedure, the received signal $r_{k}\left(n\right)$ can be expressed as

(2) $$r_{k}\left(n\right)=r_{k,t}\left(n\right)+r_{k,m}\left(n\right)+r_{k,c}\left(n\right)$$

where $r_{k,t}\left(n\right)$ is the discrete signal from the target, $r_{k,m}\left(n\right)$ is the discrete signal due to multipath and $r_{k,c}\left(n\right)$ is the discrete signal due to static clutter and noise. *k* denotes the sequence index of the received signal of frames (i.e., slow time) and *n* represents the range bin index inside a frame (i.e., fast time).

To remove the effect from clutter reflection, an average filtering has been adopted as

(3) $$c_{k}\left(n\right)=r_{k}\left(n\right)-y_{k}\left(n\right)$$

(4) $$y_{k+1}\left(n\right)=\alpha y_{k}\left(n\right)+\left(1-\alpha \right)r_{k}\left(n\right)$$

where $c_{k}\left(n\right)$ represents the cleaned received signal after clutter suppression, $y_{k}\left(n\right)$ represents the estimated clutter signal, and α∈[0,1] is the update coefficient. This recursive averaging acts as a low-pass filter along the slow-time dimension, effectively estimating and subtracting the static background. A relatively high α is adopted in the current implementation to robustly suppress strong static reflections, while remaining sensitive to human micro-motions. Tuning α to enable stationary person detection is reserved for future research.

### 3.2. Doppler and Range Spectrogram

In this work, both the Doppler and range signatures are extracted for people counting. After the clutter suppression, Doppler and range signatures are extracted with a 2-dimensional (2D) FFT on *m* of $c_{k}\left(n\right)$ from each receiver as

(5) $$\begin{array}{c} \begin{array}{cc} x_{1}\left[k,l\right]= & \frac{1}{\sqrt{N}}\sum_{n=1}^{N-1}(\frac{1}{\sqrt{M}}\sum_{m=1}^{M-1}C_{1}\left[m,n\right]\\ \; & exp^{-j2\pi mk/N})exp^{-j2\pi nl/N} \end{array} \end{array}$$

(6) $$\begin{array}{c} \begin{array}{cc} x_{2}\left[k,l\right]= & \frac{1}{\sqrt{N}}\sum_{n=1}^{N-1}(\frac{1}{\sqrt{M}}\sum_{m=1}^{M-1}C_{2}\left[m,n\right]\\ \; & exp^{-j2\pi mk/N})exp^{-j2\pi nl/N} \end{array} \end{array}$$

(7) $$\begin{array}{c} \begin{array}{cc} x_{3}\left[k,l\right]= & \frac{1}{\sqrt{N}}\sum_{n=1}^{N-1}(\frac{1}{\sqrt{M}}\sum_{m=1}^{M-1}C_{3}\left[m,n\right]\\ \; & exp^{-j2\pi mk/N})exp^{-j2\pi nl/N} \end{array} \end{array}$$

where *M* denotes the number of frames (slow-time dimension) and *N* denotes the number of samples per chirp (fast-time dimension). Matrices $C_{1}$, $C_{2}$, and $C_{3}$ are the clutter-suppressed time-domain data for the three receiving channels, with dimensions M×N. Results $x_{1}$, $x_{2}$, and $x_{3}$ are the 2D range-Doppler maps for the respective receivers.

To extract discriminative features, we first identify the strongest peak in the aggregated range-Doppler map, with its row and column indices denoted as (K,L). To construct the Doppler signature, we extract the entire *K*-th row from each range-Doppler map:

(8) $$\begin{array}{c} d_{1}=x_{1}\left[K,:\right] \end{array}$$

(9) $$\begin{array}{c} d_{2}=x_{2}\left[K,:\right] \end{array}$$

(10) $$\begin{array}{c} d_{3}=x_{3}\left[K,:\right] \end{array}$$

where $d_{1}$, $d_{2}$, and $d_{3}$ are 1-dimensional vectors representing the Doppler signatures extracted from $x_{1}$, $x_{2}$, and $x_{3}$, respectively.

Similarly, to construct the range signature, we extract the entire *L*-th column:

(11) $$\begin{array}{c} r_{1}=x_{1}\left[:,L\right] \end{array}$$

(12) $$\begin{array}{c} r_{2}=x_{2}\left[:,L\right] \end{array}$$

(13) $$\begin{array}{c} r_{3}=x_{3}\left[:,L\right] \end{array}$$

**Figure 2:**
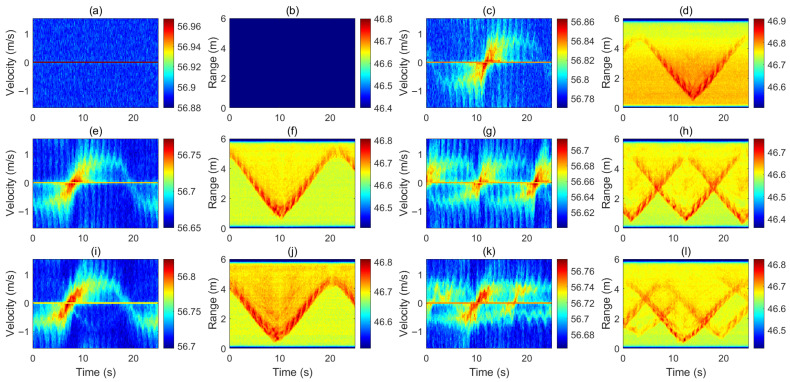
Doppler and range spectrogram measured from each motion state: (**a**) no-person Doppler, (**b**) no-person range, (**c**) one person walking Doppler, (**d**) one person walking range, (**e**) two persons (same direction) Doppler, (**f**) two persons (same direction) range, (**g**) two persons (toward each other) Doppler, (**h**) two persons (toward each other) range, (**i**) three persons (same direction) Doppler, (**j**) three persons (same direction) range, (**k**) three persons (random walking) Doppler, (**l**) three persons (random walking) range.

Figure 2 shows the Doppler and range spectrograms for 0–3 people. The single-person case (Figure 2c,d) exhibits a sinusoid-like Doppler trace and a rounded V-shaped range curve, driven by turning-point deceleration/acceleration and arm-swing micro-motion superimposed on near-constant walking speed. The two-person case (Figure 2g,h) shows two partially overlapping Doppler/range tracks, indicating simultaneous targets at different ranges/velocities. The three-person case (Figure 2k,l) presents multiple intersecting tracks, reflecting increased multi-target complexity. The no-person case (Figure 2a,b) contains only background noise, confirming no motion.

### 3.3. Data Augmentation for Robustness

Training a deep neural network, such as ResNet-50, typically requires a large and diverse dataset to achieve strong generalization. However, given the limited number of volunteers and the constrained data collection duration in this study, we implemented data augmentation techniques to artificially expand and diversify the dataset, thereby improving the model’s robustness and adaptability.

To emulate real-world variations in radar returns, multiple augmentation strategies were applied to the range–Doppler spectrograms. These included time-reversal (temporal mirroring) to simulate motion in opposite directions, and multi-scale windowing, where frame lengths were dynamically compressed from 30 to 60 frames, allowing the model to adapt to walking speed variations. In addition, contrast adjustment within a range of ±20% was used to simulate different reflection intensities caused by varying target distances or environmental conditions.

To enhance anti-noise capability, we added additive Gaussian noise (σ=0.05, where σ denotes the standard deviation relative to normalized spectrogram amplitude) and 3 × 3-kernel Gaussian blur to simulate signal degradation and multipath interference. Given the radar’s distance resolution (3.75cm) and speed resolution (0.11m/s, Table 2), the blur kernel spans 11.25cm (distance) and 0.33m/s (speed), simulating moderate target feature diffusion. This optimizes the model for low-SNR deployment, and the combined augmentations yield a comprehensive, balanced dataset that sustains the neural network’s high accuracy across diverse indoor scenarios.

**Figure 3:**
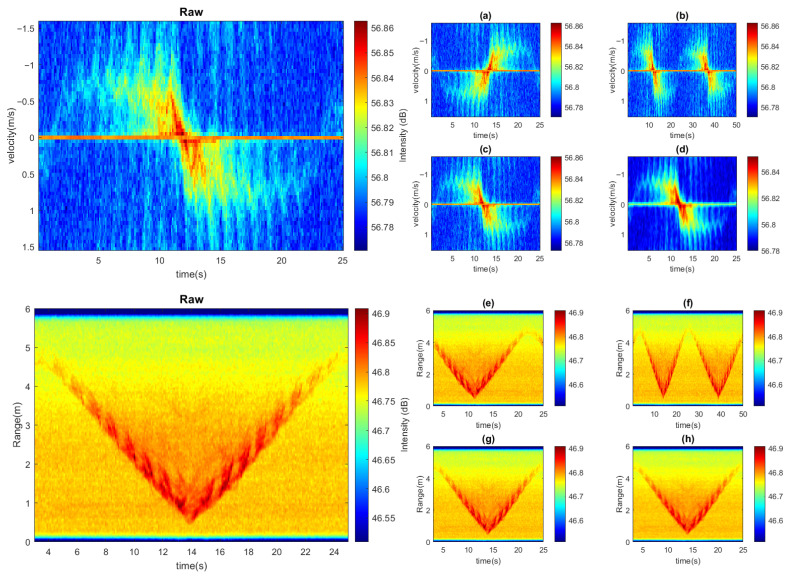
The data augmentation and imaging blurring: Range data: (**a**) mirror image, (**b**) twice the length of the time window, (**c**) Gaussian blur σ=0.4, (**d**) Gaussian blur σ=0.8; Doppler data: (**e**) mirror image, (**f**) twice the length of the time window, (**g**) Gaussian blur σ=0.4, (**h**) Gaussian blur σ=0.8.

The overall augmentation process effectively increased the usable data size by incorporating the transformed samples into the training and validation sets. As illustrated in Figure 3, if the original dataset size is *D*, an augmentation ratio *a* expands the dataset into aD. This augmentation pipeline substantially improved both the robustness and generalization of the proposed mmWave radar-based people counting system.

### 3.4. Model Configuration

The selection of ResNet-50 [22] was motivated by its proven capability in learning complex representations, which is essential for interpreting subtle Doppler-time and range–time patterns in radar-based human sensing tasks. The deep residual architecture of ResNet enables efficient gradient propagation and facilitates the extraction of multi-scale motion and spatial features.

As a representative ResNet variant, it balances feature capacity and computational efficiency. It outperforms shallow networks in pattern discrimination, while avoiding the high complexity of deeper variants. This makes it a practical fit for real-time indoor monitoring.

Each spectrogram captures complementary motion characteristics: the range–time spectrogram reflects the target’s spatial displacement, while the Doppler–time spectrogram encodes velocity and micro-motion information. These two inputs are first passed through independent convolutional branches, each consisting of convolution, batch normalization, and ReLU activation layers to extract low-level spatial and temporal features. The resulting feature maps are then concatenated along the channel dimension and fed into the main ResNet-50 backbone for hierarchical feature fusion and joint analysis. This dual-branch architecture enables the model to exploit both range and velocity information simultaneously.

In the training phase, our objective was to minimize the discrepancy between the one-hot encoded labels for each instance and the predicted probability distribution. The loss function, a key metric in this process, is defined using the Cross-Entropy Loss as follows:

(14) $$\mathrm{Loss}=-\frac{1}{N}\sum_{i=1}^{N}\sum_{c=1}^{C}y_{ic}\log\left({\hat{y}}_{ic}\right)\;$$

where *N* represents the total number of training instances, *C* denotes the number of classes, $y_{ic}$ represents the actual label of the i-th instance for class c, and ${\hat{y}}_{ic}$ symbolizes the predicted probability of the *i*-th instance for class c. Table 3 summarize the training parameter used in this work.

## 4. Experimental Setup

This study aims to classify both the number of personnel (0–3 people) and their motion states in indoor environments using the proposed concepts and a 60 GHz mmWave radar sensor. The overall experimental process includes three core stages: radar data acquisition, signal processing and dataset construction, and model training and evaluation. The evaluation phase includes standard performance testing as well as specialized experiments for data augmentation and model robustness (e.g., Gaussian blur perturbation).

**Figure 4:**
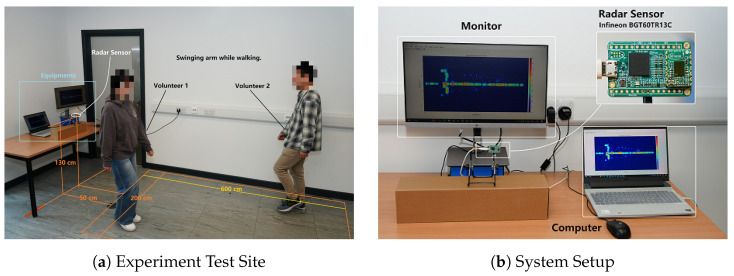
Schematic diagram of radar experimental equipment: (**a**) Indoor layout diagram of the radar sensor, (**b**) Installation settings of the data acquisition equipment.

Experiments were conducted in a standard indoor environment with office settings as background clutter. The mmWave radar sensor was mounted at a height of 1.3 m, facing an open area to cover the entire motion zone. A rectangular region, centered 0.5 m in front of the radar, with a 2 m width and a 6 m depth, was designated as the active movement area. During each trial, volunteers performed predefined motion activities within this region, as illustrated in Figure 4a.

Figure 4b shows the setup of the mmWave radar sensor. The radar data were transmitted to a computer through a USB cable. The processed data were then displayed on the monitor and stored locally.

The 60 GHz Infineon BGT60TR13C [21] radar sensor employed in this study operates in the 60 GHz ISM frequency band, with a center frequency of 59 GHz. Equipped with a 4 GHz bandwidth, the radar achieves a range resolution of 3.75 cm and a velocity resolution of 0.11 m/s, enabling precise detection of target positions and motion dynamics. The sensor adopts a 1 Tx (transmit) and 3 Rx (receive) antenna configuration. Data acquisition is performed at a sampling rate of 1.2 MS/s. Detailed technical parameters of the radar system are summarized in Table 2.

Both the Doppler and range spectrogram were processed in real time on the laptop. They were also timestamped for synchronization and labeled for machine learning. There are a total of six different motion patterns that were conducted. Including no person, one person walking, two persons walking in the same direction, two persons walking toward each other, three persons walking in the same direction, and three persons walking in a random direction. Each motion pattern had a total acquisition duration of around 120 s. The detailed duration and valid frame counts for each motion pattern are summarized in Table 4, including the size of measurement, division and status of both the raw data and the augmented data set. The corresponding range and Doppler spectrogram are shown in Figure 2.

Subsequently, the dataset of all six motion patterns was divided into a training set (including a validation set) and an independent test set. To enhance the model’s generalization ability and mitigate overfitting issues that may arise from small-sample data, data augmentation techniques were applied to the training set. The specific methods are detailed in Section 3.

## 5. Experimental Results

The classification results are presented in this section. Both range and spectrogram data were passed into the ResNet-50 model for training purposes with data augmentation and mirroring. Afterwards, testing data was used for validation.

### 5.1. Performance of Model

**Figure 5:**
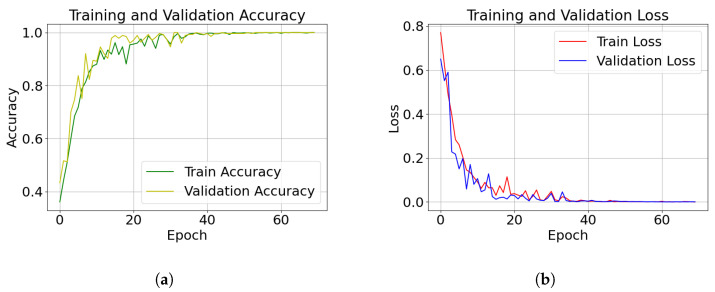
Training process curves of the model: (**a**) training/validation accuracy curves; (**b**) training/validation loss curves.

The variation patterns of accuracy and loss during the model training phase are shown in Figure 5, which contains two subfigures corresponding to the accuracy curves and loss curves, respectively.

Figure 5a shows the training and validation accuracy over different epoch. As can be seen, both the training set and validation set accuracies increase rapidly with the number of iterations, and stabilize after 30 iterations. Loss curves are presented in Figure 5b. Both the training and validation set losses exhibit an overall decreasing trend with the number of iterations, accompanied by minor local fluctuations, and approach 0 after 30 iterations. Analyzing the above two results, the difference in accuracy between the training set and the validation set is still relatively small, and the accuracy of the validation set converges to 1. This indicates that the model does not have a significant overfitting problem and has good convergence and a stable training process.

### 5.2. Motion Pattern and People Counting Analysis

**Figure 6:**
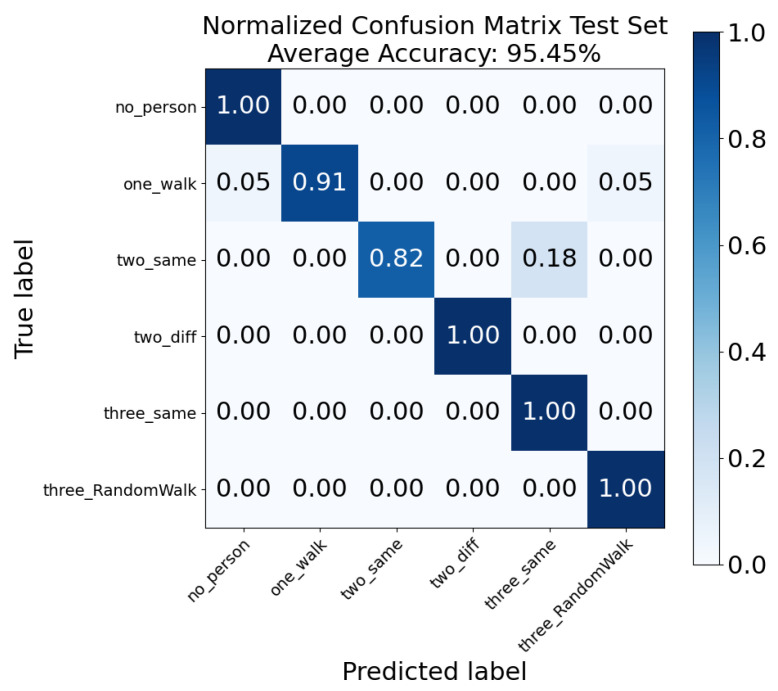
Normalized confusion matrix for the “0–3 people + 6 motion states” joint classification task.

Figure 6 demonstrates the confusion matrix of six motion patterns. As can be seen, the average test set accuracy of this task is 95.45%. Most of the motion patterns reached 100% accuracy, including no person, two persons (different direction), three persons (same direction) and three persons (random walk). The lowest accuracy is when there are two persons (same direction), at 82%, with 18% misclassified as three persons (same direction). This is because both the range and Doppler spectrogram are with similar shapes for these two patterns due to superposition, where the only difference is due to the received power. Also, the accuracy of the one-person walk category, which is 0.91, with 5% misclassified as no person and 5% misclassified as three (random walk). This is believed to be due to the external interference during the data collection. As one person walks, they should have a significantly different pattern than no person or three persons walking.

**Figure 7:**
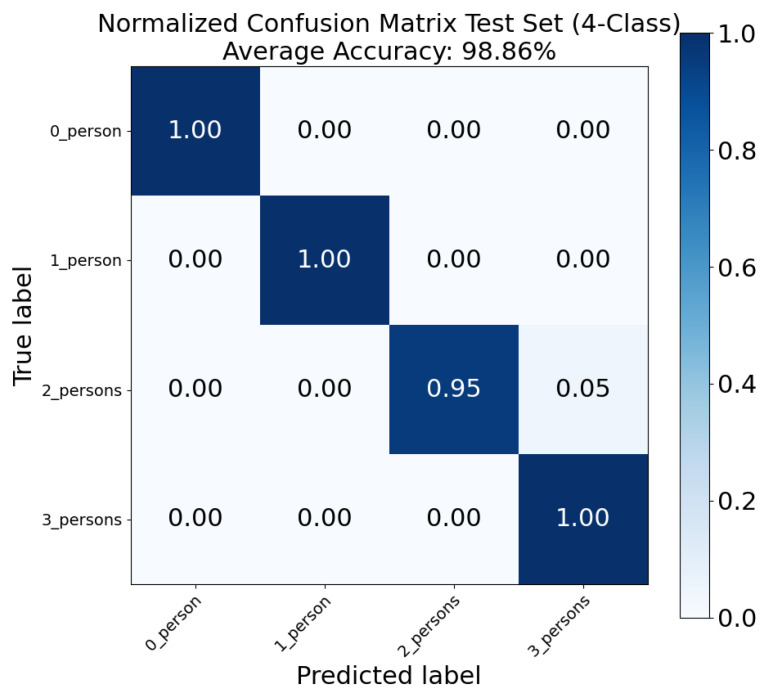
Normalized confusion matrix for the single 0–3 people counting task.

Afterwards, we want to check the accuracy when counting different numbers of people. The dataset was then rearranged as 0 person, 1 person, 2 persons, and 3 persons. The corresponding classification performance is shown in Figure 7. As can be seen, the average test set accuracy of this task is 98.86%. Noticeably, the classification accuracy of 0 person, 1 person, and 3 persons reached 100%. The accuracy of two persons is 95%, with only 5% misclassified as three persons. This indicates that the proposed use of both range and Doppler spectrograms can deliver reliable people counting when the number of persons is low. It is also expected that the accuracy will remain sufficient when more persons are involved. Notably, this 98.86% counting accuracy outperforms the 71% accuracy reported in [15] and the 85.6% accuracy from [14], both of which are 60 GHz mmWave radar-based people counting solutions.

### 5.3. Data Augmentation Effectiveness Analysis

This analysis aims to explore the necessity and effectiveness of data augmentation techniques used in this paper to improve the performance of the model. This is to reflect the situation where the collected dataset is limited. Raw range and Doppler spectrograms were selected as the benchmark. A series of datasets with different augmentation ratios were generated by systematically applying the data augmentation methods described in Section 3, including time reversal, signal amplitude scaling, and micro-Doppler feature translation. The augmentation ratios were defined as follows: α∈0.2k∣k=0,1,…,5.

**Figure 8:**
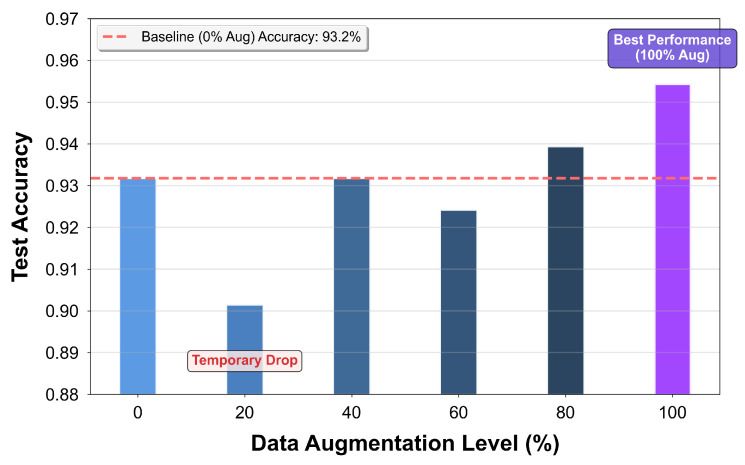
Model test accuracy variation under different data augmentation ratios.

Afterwards, the model was trained and tested on each dataset, and the comparison of test accuracies is shown in Figure 8. The baseline test accuracy is with 0% augmentation, which reaches 93.2%. At a 20% augmentation ratio, a temporary accuracy drop occurs due to a slight imbalance in data distribution, which reduces model learning stability. At 40% augmentation, the accuracy returns to the baseline level. It then experiences a minor dip between 40% and 60%. From 60% to 100% augmentation, accuracy exhibits a clear monotonic upward trend, reaching a peak of approximately 95.5% at 100%. This indicates that the model still has good classification capability even with only 120 s of raw data collected. Moreover, data augmentation can further improve model performance, especially at high augmentation ratios, verifying the necessity of data augmentation.

### 5.4. Model Anti-Interference Analysis

**Figure 9:**
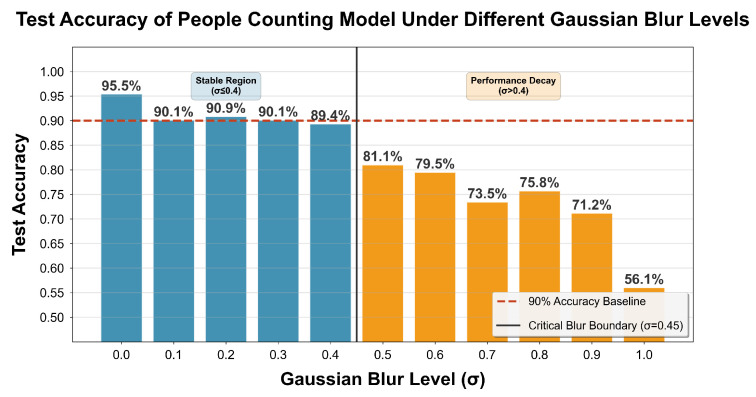
Model test accuracy variation under different Gaussian blur coefficients.

This experiment evaluates the model’s robustness against degraded input signal quality, simulating low signal-to-noise ratio (SNR) or low-resolution conditions caused by signal attenuation, multipath effects, and other factors in real indoor environments. Gaussian blur was applied to radar micro-Doppler feature maps to simulate low signal-to-noise ratio (SNR) or low-resolution scenarios caused by signal attenuation and multipath interference in real environments. The variation in the model’s test accuracy with different Gaussian blur coefficients σ is shown in Figure 9, where σ∈{0.1k∣k=0,1,…,10}.

As the figure presents, when σ≤0.4, the feature maps remain in a stable region with only slight blurring. The model’s test accuracy stays between 89.4% and 95.5%, indicating minor performance degradation. When σ>0.4, the model enters a performance decay region where feature obscuration increases significantly. The accuracy drops rapidly to 81.1% at σ=0.5 and 56.1% at σ=1.0. Using 90% accuracy as a baseline, the critical blur boundary is determined as σ=0.45. The model demonstrates reliable robustness up to a blur coefficient of σ=0.4, effectively handling most indoor low-SNR scenarios.

### 5.5. Application Potential

The proposed 60 GHz mmWave radar system demonstrates strong potential for deployment in smart homes and intelligent indoor environments. It can deliver accurate, real-time occupancy perception, which is essential for multiple applications. Thanks to its high range resolution (3.75 cm) and fine Doppler sensitivity, the system can reliably distinguish subtle motion patterns and accurately count individuals, achieving 98.86% accuracy in people counting and 95.45% in motion-state classification. Such performance suggests that the system can be integrated into scenarios where privacy-preserving sensing is preferred over camera-based solutions, including adaptive lighting and heating control, intrusion detection, fall detection assistance, and elderly care monitoring.

Another advantage supporting practical deployment is the radar’s effective sensing coverage of up to 8 m, enabling a single sensor to cover typical living spaces such as living rooms, corridors, or bedrooms. Its robustness to environmental noise, maintaining stable accuracy under Gaussian blur up to σ = 0.4 (as shown in Figure 9). This further indicates reliable performance under low-SNR, cluttered, or multipath-rich indoor conditions commonly seen in real homes.

However, the current system is evaluated primarily on controlled indoor layouts and a limited population size. Performance in more complex home environments, like more furniture arrangements, dense multipath, and highly random multi-person interactions, may differ from current settings. In addition, only 0–3 person scenarios were studied; thus, higher-occupancy conditions require further validation. Moreover, real-time embedded deployment also demands optimization of computational load beyond the current laptop-based processing. For example, a Raspberry Pi-based processing platform would significantly improve its deployability.

## 6. Conclusions

This study develops an indoor people counting system integrating a 60 GHz radar sensor and an improved ResNet-50 model, with experimental evaluations focusing on basic performance, impact on data volume, and robustness. In terms of basic capability, the system achieves a 95.45% average accuracy in the six motion patterns classification and a 98.86% average accuracy in the single 0–3 people counting task. Regarding data volume sensitivity, the system shows good adaptability to limited data scenarios, and data augmentation further enhances its generalization ability. For robustness, the system demonstrates stable performance under mild Gaussian blur interference, indicating its adaptability to most common indoor signal conditions. Overall, the proposed system meets the practical requirements for indoor people counting and motion state recognition, providing a reliable solution for indoor human activity perception.

Future work will focus on improving the system’s generalization and readiness for real-world deployment. This includes expanding the dataset to cover more diverse indoor environments, furniture layouts, and complex multi-person interactions, and additional human activity types beyond the six patterns in this study. Also, it is worth exploring lighter or attention-based architectures to further enhance robustness while reducing computational cost. Practical embedded implementation will also be investigated to achieve real-time operation on edge devices rather than on a laptop. Finally, long-term deployment tests and integration with smart-home or IoT platforms, such as energy management, fall detection, and elderly care systems, will be conducted to evaluate the system’s performance under realistic, everyday conditions.

## Figures and Tables

**Table 1:** Comparative analysis of radar-based people counting systems.

Source	Resolution	Radar Hardware	Hardware Specification	Signal Processing	Performance
[19]	10.7 cm	Novelda X4M03 UWB	1 Tx × 1 Rx; Carrier Frequency = 7.29 GHz, Bandwidth = 1.4 GHz	Bi-Motion-Model-Framework (BMMF): SVM classifies motion states (static/moving) → Probabilistic Model/Convolutional Neural Network counts people	0–6 people: 98.8% (±1, static), 95.5% (moving); 7–26.3% accuracy gain vs. probabilistic model/CNN; mitigates multipath-induced fluctuations
[17]	Not specified	NVA-R661 IR-UWB	Not specified; Carrier Frequency = 6.8 GHz, Bandwidth = 2.3 GHz	Improved AlexNet Convolutional Neural Network (data-driven, no manual threshold; clutter removal is harmful)	0–3 people: 99.9% (queue, no occlusion), 99.05% (confined room); resolves queue blocking, avoids info loss in traditional signal processing
[15]	15 cm	Infineon BGT60TR13C	1 Tx × 3 Rx; Carrier Frequency = 61 GHz, Bandwidth = 1 GHz	Knowledge Distillation + ResBlock (uses distilled camera data for generalization)	0–6 people: 89% (same sensor), 71% (different); Enhances generalization across sensor positions
[13]	10 cm	Novelda X4M03 IR-UWB	1 Tx × 1 Rx; Carrier Frequency = 7.29 GHz, Bandwidth = 1.5 GHz	Modified CLEAN (MD-CLEAN) + Multi-Threshold + Random Forest + Principal Component Analysis (dim. reduction to 15D)	0–10 people: 92.83% (strict accuracy); 11.89% higher than CT-based method; Solves energy saturation in wide areas, Multi-Threshold improves feature separability
[14]	7.3 cm	TI IWR6843AOP	3 Tx × 4 Rx; Carrier Frequency = 60 GHz, Bandwidth = 3.6 GHz	Space Boundary-Aware People Tracking and Counting (SBA-PTC): data screening (0/1 person) → boundary estimation → EKF+DBSCAN tracking/counting	1–4 people: 85.63% counting accuracy, tracking MAE 0.071 m; adaptive boundary estimation removes multipath/shadow ghosts without prior environment info
[20]	60 cm	Joby (former INRAS) RadarBook2 (RBK2)	2 Tx × 8 Rx; Carrier Frequency = 24 GHz, Bandwidth = 250 MHz	Grouped target tracking + seamless counting: EKF+GNN association+DBSCAN clustering → SVM (RA spatial features + MODWT Doppler features);	0–4 people: 93.15% counting accuracy (median-filtered), tracking OSPA = 0.335; robust to indoor clutter/multipath, adapts to stop–go and direction changes
Our Work	4.7 cm	Infineon BGT60TR13C	1 Tx × 3 Rx; Carrier Frequency = 59 GHz, Bandwidth = 4 GHz	ResNet50 (input data type: to be supplemented, e.g., range-Doppler map)	To be supplemented (e.g., 95.45% (Enclosed Space) + Mean Absolute Error/±1 error; Adapts to 6 motion modes for robustness)

**Table 2:** FMCW radar specifications.

Parameter	Value
Chip model	Infineon BGT60TR13C
Center frequency	59 GHz
Bandwidth	4 GHz
Sampling rate	1.2 MS/s
Antenna configuration	1 Tx, 3 Rx
Modulation type	FMCW
Operating band	60 GHz ISM
Range resolution	3.75 cm
Velocity resolution	0.11 m/s

**Table 3:** Model training configuration parameters.

Parameter	Value
Batch Size	8
Total Epochs	70
Optimizer	AdamW
Initial Learning Rate ($\eta_{\max}$)	$4.8\times 10^{-4}$
Minimum Learning Rate ($\eta_{\min}$)	$4.8\times 10^{-5}$
Weight Decay	0.05
Warm-up Epochs	5
Dropout Rate	0.2

**Table 4:** Dataset composition by motion pattern.

State	Total Meas. (F × D × R)	Raw Training Images (Count)	Augmented Training Samples (Count)	Test Images (Count)	Duration (s)
0	1203 × 64 × 640	108	864	20	120.3
1	1063 × 64 × 640	96	768	20	106.3
2S	1061 × 64 × 640	96	768	20	106.1
2O	1067 × 64 × 640	96	768	20	106.7
3S	1209 × 64 × 640	108	864	20	120.9
3R	1202 × 64 × 640	108	864	20	120.2

*Note*: 0 = no personp; 1 = 1 person walking; 2S = 2 persons (same direction); 2O = 2 persons (opposing directions); 3S = 3 persons (same direction); 3R = 3 persons (random walking); Total Meas. = total measurements; F = frame; D = Doppler; R = range.

## Data Availability

The data presented in this study are available on request from the corresponding author due to privacy restrictions.
